# Evolution and diversity of inherited viruses in the Nearctic phantom midge, *Chaoborus americanus*

**DOI:** 10.1093/ve/veac018

**Published:** 2022-03-10

**Authors:** Matthew J Ballinger, Rebecca C Christian, Logan D Moore, Derek J Taylor, Afsoon Sabet

**Affiliations:** Department of Biological Sciences, Mississippi State University, PO Box GY, Mississippi, MS 39762, USA; Department of Biological Sciences, Mississippi State University, PO Box GY, Mississippi, MS 39762, USA; Department of Biological Sciences, Mississippi State University, PO Box GY, Mississippi, MS 39762, USA; Department of Biological Sciences, The State University of New York at Buffalo, 109 Cooke Hall, Buffalo, NY 14260, USA; Department of Biological Sciences, Mississippi State University, PO Box GY, Mississippi, MS 39762, USA

**Keywords:** insect-specific viruses, persistent infection, vertical transmission, symbiosis, phylogeography, RNA interference

## Abstract

Inherited mutualists, parasites, and commensals occupy one of the most intimate ecological niches available to invertebrate-associated microbes. How this transmission environment influences microbial evolution is increasingly understood for inherited bacterial symbionts, but in viruses, research on the prevalence of vertical transmission and its effects on viral lineages is still maturing. The evolutionary stability of this strategy remains difficult to assess, although phylogenetic evidence of frequent host shifts and selective sweeps have been interpreted as strategies favoring parasite persistence. In this study, we describe and investigate a natural insect system in which species-wide sweeps have been restricted by the isolation of host populations. Previous work identified evidence of pronounced mitochondrial genetic structure among North American populations of the phantom midge, *Chaoborus americanus*. Here we take advantage of the geographical isolation in this species to investigate the diversity and persistence of its inherited virome. We identify eight novel RNA viruses from six families and use small RNA sequencing in reproductive tissues to provide evidence of vertical transmission. We report region-specific virus strains that mirror the continental phylogeography of the host, demonstrating that members of the inherited virome have independently persisted in parallel host lineages since they last shared a common ancestor in the Mid-Pleistocene. We find that the small interfering RNA pathway, a frontline of antiviral defense in insects, targets members of this inherited virome. Finally, our results suggest that the Piwi-mediated RNA silencing pathway is unlikely to function as a general antiviral defense in *Chaoborus*, in contrast to its role in some mosquitoes. However, we also report that this pathway generates abundant piRNAs from endogenous viral elements closely related to actively infecting inherited viruses, potentially helping to explain idiosyncratic patterns of virus-specific Piwi targeting in this insect.

## 1. Introduction

Inherited microbes are widely recognized for their contributions to the ecology and evolution of invertebrate hosts (Oliver and Martinez 2014; Hosokawa et al. 2020). Common benefits include nutritional supplementation and pathogen protection, but negative effects are also prevalent and persistent, e.g. reproductive parasitism. While bacterial taxa dominate these niches, microbial eukaryotes including protists (Ohkuma et al. 2009) and fungi (Biedermann and Vega 2020) have also transitioned into long-term symbionts of invertebrates. In contrast, the spectrum of ecological roles served by inherited viruses is poorly understood. Recent and rapid growth in the number of recognized viral taxa within invertebrate hosts—a product of broad and unbiased metagenomics sequencing surveys of field-collected hosts—reveal an ongoing ignorance of the virosphere (Li et al. 2015; Shi et al. 2016). While still rare, studies of host interactions have highlighted several unexpected viral roles, including defense (Xu et al. 2020), reproductive manipulation (Fujita et al. 2021), and even offensive alliances in hosts with parasitic lifestyles (Coffman, Harrell, and Burke 2020); however, broad evolutionary trends in host specificity, fitness effects, coinfection dynamics, and transmission modes of insect-specific viruses (ISVs) still remain to be clarified. Even easily assessed features such as host species fidelity and timescales of persistence pose challenges because traditionally informative approaches like cophylogeny are confounded by virus sweeps that can obscure genealogies (Carpenter et al. 2007).

The proliferation of inherited microbes in insects can be suppressed by the microbe itself, by the host, and by other community members. For example, the growth of some inherited hemolymph-dwelling bacteria appears to be limited by independent losses of genes required to import or metabolize trehalose, the most abundant sugar in the hemolymph (Paredes et al. 2015). Other bacterial symbionts are locally restricted by tissue-specific expression of host antimicrobial peptides (Login et al. 2011) or are suppressed by lysogenic phage infections (Weldon, Strand, and Oliver 2013). The most obvious suppressor of inherited viral infections in insects is RNA silencing via RNA interference (RNAi; reviewed in Schuster, Miesen, and van Rij 2019). Here, small interfering RNAs (siRNAs) 21 nucleotides (nt) in length are created by the double-stranded RNA sensor and endonuclease Dicer-2 (Carthew and Sontheimer 2009). A single-stranded RNA endonuclease, Argonaute 2, uses virus-derived siRNAs to guide the RNAi-induced silencing complex (RISC) to identify viral RNAs through Watson–Crick base pairing and cleaves them (Rand et al. 2004; Kawamura et al. 2008). In *Aedes* mosquitoes, a second RNAi pathway mediated by Piwi family argonautes generates abundant small RNAs 25–30 nt in length from virus targets (Miesen, Joosten, and van Rij 2016). While in most arthropods the chief role of piwis is in transposon suppression (Lewis et al. 2018), the expansion of this pathway’s role into antiviral defense in mosquitoes is associated with repeated duplication of piwi endonucleases into gene families composed of 7–9 members (Morazzani et al. 2012; Schnettler et al. 2013). However, the evolution, specificity, and biological importance of this role are not yet understood. For example, mosquito genera lacking the duplicated piwis still show piwi-mediated responses to some, but not all, of their persistent viruses (Belda et al. 2019). Characterizing the relative contributions of the siRNA and PIWI-interacting RNA (piRNA) pathways in fly families related to mosquitoes, including the phantom midges (Chaoboridae) and the frog-biting midges (Corethrellidae) may lend insight into their evolution as mediators of virus infection.

The phantom midge, *Chaoborus americanus*, is a key invertebrate predator of fishless fresh waterbodies (von Ende 1979). While the species has a flighted adult stage and broad Nearctic distribution, potential dispersal barriers, e.g. dense forest, have been suggested to limit migration into some parts of the range (Borkent 1981). We previously identified a novel phasmavirid in *C. americanus* and reported that two distinct regional clades could be resolved using mitochondrial and viral loci, supporting the existence of dispersal barriers and suggesting a long-term virus–host association (Ballinger and Taylor 2019). In the current study, we use field collections, phylogenetics, RNASeq, and tissue-specific small RNA sequencing to identify novel viruses and estimate the timescales of their persistent infections in *C. americanus*. We discovered eight novel RNA viruses, all with either negative sense or double-stranded RNA genomes. Six infect the eggs or ovaries and persist across isolated sampling locations including Alaska, the Pacific Northwest, and the Midwestern United States. Dispersal barriers appear to have limited transmission of viruses between regionally defined mitochondrial lineages, preventing viral genotype sweeps and revealing a deep evolutionary history between host and virome. We also examine the host’s RNAi responses and here our data support a role for the siRNA pathway in silencing inherited viruses. Finally, we show that small RNAs are a valuable resource for resolving the provenance of some virus-like contigs, e.g. incomplete or otherwise defective genomes, identified in metagenomics assemblies. Our results suggest these contigs frequently correspond to nonretroviral endogenous viral elements (EVEs) derived from inherited viruses, are processed by the piRNA pathway following their acquisition, and may occasionally mediate interactions between Piwi proteins and exogenous viruses.

## 2. Results

### 2.1 Genetic isolation of *Chaoborus americanus* populations in North America

**Figure 1.**
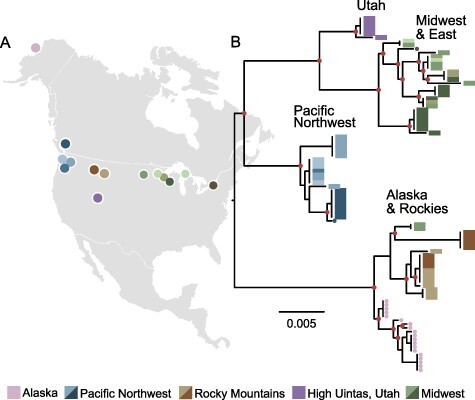
Pronounced phylogeographic structure of *Chaoborus americanus* in North America. (A) A map of North America displays collection locations of *C. americanus* populations used in this study. (B) Phylogenetic relationships of the COI barcode region. Tips are color-coded to sampling locations plotted in panel A. Tips labeled with rectangles indicate samples collected and sequenced during this study, and tips labeled with small circles indicate samples and sequences published in a previous study (Ballinger and Taylor 2019). Nodes with support values >0.8 are labeled with filled circles. This phylogeny presents a subset of a more complete tree, including support values and outgroup rooting (Supplementary Fig. S1).

We collected larval phantom midges from permanent and semi-permanent freshwater ponds across the species range. We sampled fifty-nine locations and identified sixteen ponds with larval *C. americanus* in the Western and Midwestern United States and Canada, including Michigan, Wisconsin, Minnesota, Montana, Utah, Idaho, Washington, and British Columbia (Fig. 1A; Supplementary Table S1). We sequenced the mitochondrial cytochrome oxidase subunit I (COI) barcode locus from each population and found distinct genetic clades defined by sampling location (Fig. 1B). Despite the phantom midge’s flighted adult stage, we found little evidence of mitochondrial gene flow between regions—just two individuals collected in Minnesota were harboring a genotype similar to those in Montana and Idaho (Fig. 1B). To increase sample size and improve resolution, we also analyzed all *C. americanus* sequence entries in the Barcode of Life Database (BOLD), which add considerable representation in the Midwest and East, while continuing to support near complete genetic isolation among regions (Supplementary Fig. S1). Two observations support the interpretation that isolation is not due to geographic distance alone. First, the region covered by the Midwest and East is expansive yet contains a weak genetic structure. Second, the genotypes from the Rocky Mountains region group with Alaskan genotypes rather than those in nearby Western British Columbia and Washington. Meanwhile, the Utah populations are closest in proximity to Montana and Idaho, yet these are among the most divergent pairwise genetic comparisons in our dataset. Together, these observations suggest physical barriers or habitat deserts have limited the dispersal of *C. americanus* across the North American continent, resulting in long-term maintenance of isolated mitochondrial lineages.

### 2.2 Genomic characteristics of novel phantom midge RNA viruses

**Figure 2.**
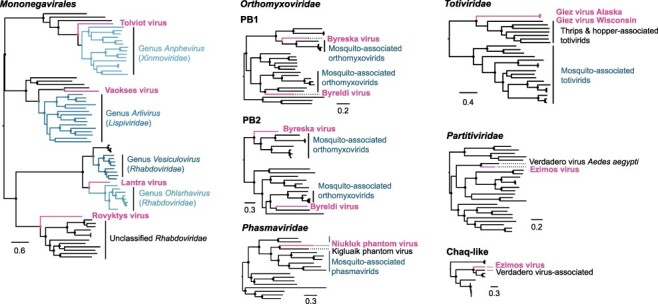
Evolutionary relationships of *Chaoborus* viruses. Phylogenetic analyses of novel virus sequences indicate the virome of *Chaoborus* is composed of divergent RNA virus taxa. Phylogenies are midpoint rooted and labeled with taxonomic family, except for members of *Mononegavirales*, in which the phylogeny contains four families in addition to unassigned taxa. Tips corresponding to novel viruses are indicated in bold typeface. Phylograms were built from full-length RdRp amino acid sequence alignments unless otherwise indicated. For totivirids, the full capsid-polymerase fusion sequence was used. Nodes with FastTree maximum likelihood-like support values of 0.9 or greater are labeled with a filled circle.

To identify novel virus genomes in *Chaoborus americanus*, we sequenced ribosome-depleted total RNA libraries from eight larvae collected in British Columbia, Washington, and Wisconsin. We also searched a previously published metagenomics assembly from a pool of five larvae collected in Alaska. We identified eight novel virus genomes, summarized below (Fig. 2). All novel viruses belong to the realm *Riboviria* and encode either negative sense or double-stranded RNA genomes. Including a previously identified phasmavirid, Niukluk phantom virus, three of these viruses were present across all regions, including Alaska. Tissue sources and library types are summarized in Supplementary Table S1.

### 2.3 Negative sense RNA viruses

#### 2.3.1 Xinmoviridae

**Figure 3.**
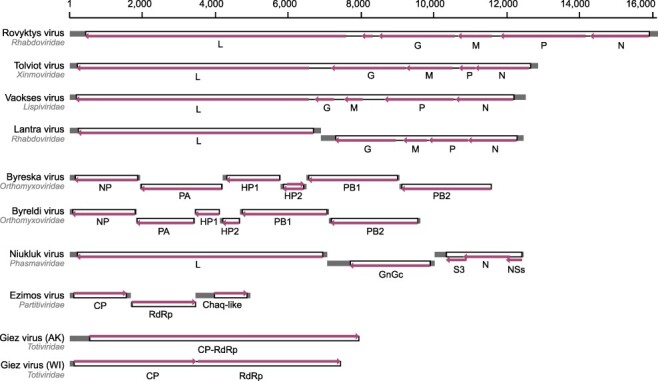
Genome sizes and structures of novel RNA viruses in *Chaoborus*. Diagrams depict genome size and predicted coding and untranslated region for eight novel viruses and one described previously (Niukluk virus). The scale bar indicates genome length in nucleotides. Arrows indicate directionality of predicted open reading frames and gene name labels are below. Genomes of negative sense RNA viruses are shown in negative orientation, those with double-stranded RNA genomes are shown in positive orientation.

A complete xinmovirid (Order *Mononegavirales*) genome of approximately 12.7 kb was assembled from hosts in all regions. Homologs of the N, P, M, G, and L genes are present, but no putative accessory ORFs are present between these genes (Fig. 3). We propose the name Tolviot virus, meaning ‘everywhere.’ See methods for details of etymology. Phylogenetic analysis shows Tolviot virus is sister to a monophyletic clade of mosquito-infecting anpheviruses (Fig. 2) (Parry and Asgari 2018).

#### 2.3.2 Lispiviridae

Genomes of a lispivirid (Order *Mononegavirales)* assembled as single contigs from hosts in British Columbia and Washington. The two genomes share 96.8 per cent nucleotide identity and the RdRps share 37 per cent amino acid identity with the most closely related viruses in public databases. Like the xinmovirid, all five genes characteristic of members of the Order *Mononegavirales* are present and no putative accessory ORFs >50 amino acids in length were identified (Fig. 3). Phylogenetic analysis shows this virus is most closely related to Canya virus, sequenced from *Culex tarsalis* and sister to a clade of mosquito-infecting members of the genus *Arlivirus* (Fig. 2). We propose the name Vaokses virus, meaning ‘spider,’ for which the genus is named.

#### 2.3.3 Rhabdoviridae

**Figure 4.**
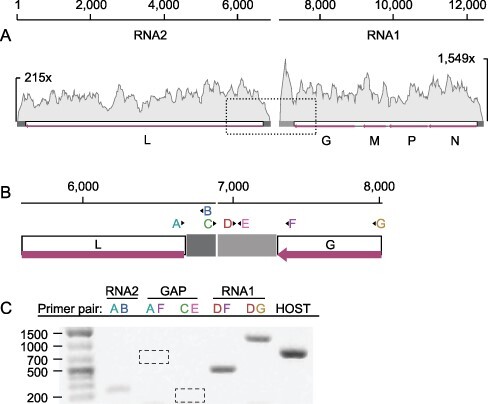
Segmented genome architecture of a novel rhabdovirid. (A) Observed pattern of genome segmentation as recovered from two independent *de novo* metatranscriptome assemblies. Naming for putative segments corresponds to conventions for distantly-related plant rhabdovirids with segmented genomes. Plotted on each segment is coverage depth of 150 bp reads. (B) PCR primer design schematic to test for continuity between the two putative segments. Letters above the L and G gene maps and UTRs indicate primer binding locations and directionality. (C) Amplification results confirm primer pair success and the presence of RNA molecules attributed to RNA1 and RNA2, but both primer pairs spanning the gap failed to produce amplicons. Dashed boxes indicate expected amplicon positions, if present.

A rhabdovirid (Order *Mononegavirales*) was assembled from two read sets acquired from one site in Washington and from Utah. The virus genomes each assembled into two fragments—one encoding putative N, P, M, and G genes flanked by untranslated regions (UTRs), and the other a full-length L gene with UTRs (Fig. 3). To explore whether the segmentation could be attributed to mis-assembly, we inspected mapped reads and found no evidence of extended terminal sequences on either segment despite deep coverage of both contigs in both read sets (Fig. 4A). In RNA1, the terminal G gene is followed by a ∼400 bp putative UTR, and in RNA2 an additional ∼240 bases precede the L coding region (Fig. 4). Read coverage at both gene-UTR junctions is also deep and consistent with the coverage across the rest of each segment. To confirm the assemblies, we designed a panel of PCR primers to amplify across the putative gap while controlling for cDNA quality and primer binding (Fig. 4B). Long and short products amplified from the terminal regions of each segment, but re-pairing these successful primers to amplify across the gap failed (Fig. 4C). Based on these data, we conclude this virus genome is segmented. Genome segmentation is atypical in rhabdovirids, but has been described in the plant-infecting genera *Dichorhavirus* and *Varicosavirus*. We expected the novel rhabdovirus to be most closely related to members of these genera; however, the polymerase sequence groups with strong support between *Vesiculovirus* and the mosquito-infecting genus *Ohlsrhavirus*, both encoding monopartite genomes (Fig. 2). Therefore, this virus represents an independent transition to genome segmentation in the Family *Rhabdoviridae*. We propose the name Lantra virus, meaning ‘two’.

#### 2.3.4 Unassigned mononegavirus

A fourth member of the Order *Mononegavirales* was found only in read sets from Washington. This virus belongs to an unclassified group of insect-associated rhabdovirids related to subfamily *Betarhabdovirinae* (Fig. 2). The virus shares only 29 per cent L amino acid identity with the closest relatives, which are mosquito-associated viruses in this group. While the mosquito viruses have genomes 12–14.4 kb in length, the novel virus genome is 16.1 kb, among the largest of invertebrate rhabdoviruses (Fig. 3). Because of its size, we expected to identify several putative open reading frames (ORFs) in the large intergenic regions, but found only one small ORF between G and L genes. Much of the genome size difference is accounted for in larger than typical P and L genes, and an expansive intergenic G-L region of 912 bases, which includes a putative ORF of 216 bases. We propose the name Rovyktys virus, meaning ‘bigger.’

#### 2.3.5 Orthomyxoviridae

A novel member of the Order *Articulavirales* was identified in larvae from British Columbia, Alaska, and Wisconsin. The virus is an orthomyxovirid related to members of the genus *Quaranjavirus* (Fig. 2). Because orthomyxovirids package segmented genomes, we used the amino acid sequences of the mosquito-associated Usinis virus to identify putative segments of the novel virus in our assembly. Homologous segments for six of the eight putative Usinis virus segments could be identified, consistent with many other quaranjaviruses (Fig. 3). Phylogenetic analyses revealed the novel genomes are not geographic variants but distinct viruses grouping with strong support in different clades of insect-infecting quaranjaviruses (Fig. 2). We propose the names Byreska virus and Byreldi virus, both portmanteaus of the prefix byre, meaning ‘six,’ and the host collection sites in Alaska, USA and Garibaldi, BC.

#### 2.3.6 Phasmaviridae

A member of the order *Bunyavirales*, Niukluk phantom virus (abbreviated to Niukluk virus hereafter), was identified in larvae from all regions. Previously, the complete genome of this phasmavirid was reported in Alaskan *C. americanus* and a partial sequence was reported from British Columbia (Ballinger and Taylor 2019). The genome is trisegmented, encoding the L protein and a glycoprotein on the L and M segments, respectively. The S segment encodes the nucleoprotein (N) flanked by a frameshift-encoded accessory gene on each side (Fig. 3). Phylogenetic analysis shows this and a related *Chaoborus*-infecting phasmavirus, Kigluaik phantom virus, are allied with a clade of mosquito-infecting phasmaviruses (Fig. 2).

### 2.4 Double-stranded RNA viruses

#### 2.4.1 Partitiviridae

Samples from Washington were infected with a partitivirid related to Verdadero virus of *Aedes aegypti* (Fig. 2) and more distantly, to Galbut virus of *Drosophila* (Webster et al. 2015; Cross et al. 2020). The genome is bisegmented, with RNA1 encoding an RdRp and RNA2, a capsid protein. A potential satellite RNA, originally designated Chaq virus, is frequently found associated with Galbut-like partitiviruses (Webster et al. 2015), and we identified a homologous Chaq segment in each of these read sets. We propose the name Ezimos virus, meaning ‘division,’ in reference to the etymology of family *Partitiviridae*.

#### 2.4.2 Totiviridae

Totivirids, most commonly associated with fungal hosts, have more recently been identified in insect metagenome assemblies. We identified a novel totivirid genome in read sets from all regions. Complete genomes range in size from 7.2–7.9 kb. Unlike typical totivirids, there is no frameshift between the capsid and RdRp, and in the genome from Wisconsin, the capsid region is separated from the RdRp region by in frame stop codons but no frameshift (Fig. 3). While the RdRp region appears most closely related to other insect-associated totivirids (Fig. 2), this virus is not closely allied with other insect viruses nor the fungal viruses comprising the established genera of *Totiviridae*. We propose the name Giez virus, meaning ‘together,’ an allusion to the absence of a frameshift.

### 2.5 Enrichment of 21 nt siRNAs identifies inherited viral infections in *Chaoborus*

**Figure 5.**
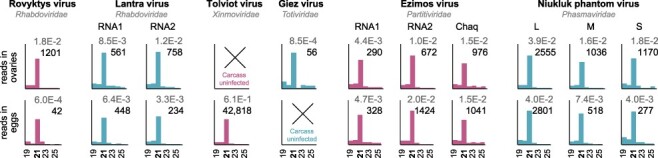
*Chaoborus* RNA viruses infect the reproductive tissues. Small RNAs sequenced from the dissected ovaries of one adult and the unfertilized dissected eggs of another and mapped to virus contigs are enriched for 21 nt fragments characteristic of the siRNA pathway. Fragment size (nt) is plotted on the *X*-axis and raw numbers of reads are plotted on the *Y*-axis. The number of 21 nt reads is shown on the right side of each graph. Normalized 21 nt read abundance is shown above, in gray, as percent of 18–31 nt reads in each sample. Tolviot virus and Giez virus were each present in only one of the two individuals sequenced. Lantra virus RNA1 encodes the N, P, M, and G genes and RNA2 encodes the L gene. Ezimos virus RNA1 encodes the RdRp and RNA2 encodes the capsid protein. Alternating bar colors are applied as a visual aid to unite multisegmented viruses.

To determine whether the metagenome-assembled virus genomes represent viruses actively infecting *Chaoborus*, we generated small RNA libraries from unmated adult females and larvae collected in Washington. From two adults, we dissected either eggs or ovaries prior to processing and constructed libraries for the carcasses and dissected tissues separately. Small RNA size profiles enriched in the 21 nt fragments produced by dipteran Dicer-2 were recovered for most of the novel viruses assembled from our larval stage RNAseq genomes. Within the libraries derived from reproductive tissues, siRNAs mapping to Tolviot, Lantra, Rovyktys, Niukluk, Giez, and Ezimos viruses are detected (Fig. 5), indicating infection by these viruses is maintained at least in part via inheritance. Tolviot virus and Giez virus were each present in only one of the two adults used to generate these libraries, and neither of the two adults were infected with Byreska, Byreldi, or Vaokses viruses. As a result, small RNA profiles for these viruses in eggs and ovaries were not assessed, but Byreska virus was present in larvae collected in Wisconsin and showed enrichment of 21 nt RNAs in these samples (Supplementary Fig. S2). Plotting siRNA mapping profiles onto each virus contig revealed uneven depth across the length of both the positive and negative sense strands for each virus (Supplementary Fig. S3). siRNAs mapping to Giez virus were extremely rare in both the ovary and carcass read sets, as well as in larval samples. This could be explained by differences in viral load or host evasion strategies. Additional sampling will support future comparative analyses of the antiviral RNAi response across viruses, as well as between *Chaoborus* and other dipteran insects.

### 2.6 Geographic isolation reveals deep history of codivergence

**Figure 6.**
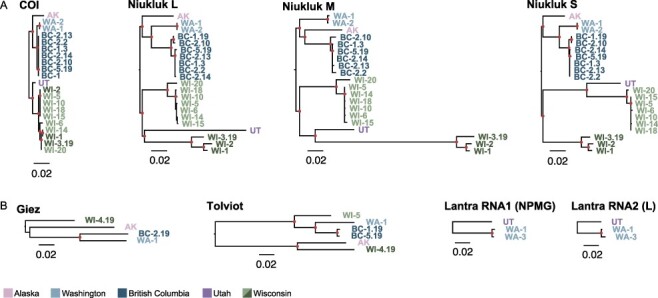
Host and virus phylogeography is consistent with long-term codivergence. (A) Phylogram of the mitochondrial COI locus for all Niukluk virus-infected hosts and each of the three virus genome segments. Trees in panel A are rooted on the AK-WA node to facilitate topological comparisons. (B) Phylograms for Giez virus, Tolviot virus, and Lantra virus also recover divergent virus strains. Trees in panel B are midpoint rooted. All phylograms are scaled to 0.02 substitutions per site to highlight contrasting genetic distances among loci. Tips are labeled with sampling location and sample ID. Nodes with FastTree maximum likelihood-like support values of one are labeled with a filled circle.

To investigate continent-wide codivergence between host and virus, we focused on one inherited virus present in all regions for targeted prevalence screens and virus sequencing. We extracted total RNA from 353 *C. americanus* larvae. Niukluk virus prevalence across the continent was 11.1 per cent and within-population infection frequency ranged from 3 to 52 per cent (Supplementary Table S3). In addition to six complete Niukluk virus genomes from RNASeq data sets, we generated partial L, M, and S segment sequences from fifteen larvae in Utah, British Columbia, and Wisconsin by Sanger sequencing. Phylogenetic analysis of the twenty-one full or partial virus genomes showed well-supported clades corresponding to host mitochondrial clades (Fig. 6A). Branching order at deeper nodes and branch length variation raise the possibility of ancient reassortment or episodic shifts in evolutionary rate, particularly within the glycoprotein-encoding M segment. A recombination analysis of the concatenated alignment identified potential breakpoints at the gene boundaries, but this analysis failed to reject evolutionary rate variation as an explanation for the identified breakpoints (Supplementary Fig. S4).

Because these data provide compelling evidence that Niukluk virus and *C. americanus* populations have been codiverging in isolated habitat islands across North America, we used the insect COI molecular clock to estimate the evolutionary timescale of persistence. Based on 2.88 per cent sequence divergence between COI alleles in Alaska and Wisconsin and a clock rate of 3.54 per cent divergence per million years (Papadopoulou, Anastasiou, and Vogler 2010), the most recent common ancestor of Niukluk virus-infected *C. americanus* populations in North America existed around the middle Pleistocene, approximately 800,000 years before present.

We also examined the genetic distance and phylogenetic relationships in the other inherited viruses that have persisted in multiple regions. In some cases, we used mapped small RNAs to reconstruct partial virus genomes from populations where RNASeq data was unavailable. We found evidence of divergent strains of these viruses persisting across the continent as well (Fig. 6B), although we cannot conclude they represent region-specific strains from the available sequences. The topology of the Giez virus phylogeny recapitulates that of the host and Niukluk virus trees, while in the Tolviot virus phylogeny, Alaskan viruses are more closely allied with Wisconsin strains than expected (Fig. 6B). Tolviot virus, like Niukluk virus, is also present as two divergent strains in Wisconsin. We note that Fig. 1 shows a mitochondrial haplotype allied with Alaskan and Rocky Mountain haplotypes was found in the Midwest at low frequency, possibly a representative of the mitochondrial lineage in which these virus strains historically diverged before a host lineage shift.

### 2.7 The *Chaoborus* piRNA pathway is minimally but differentially active on viral targets

**Figure 7.**
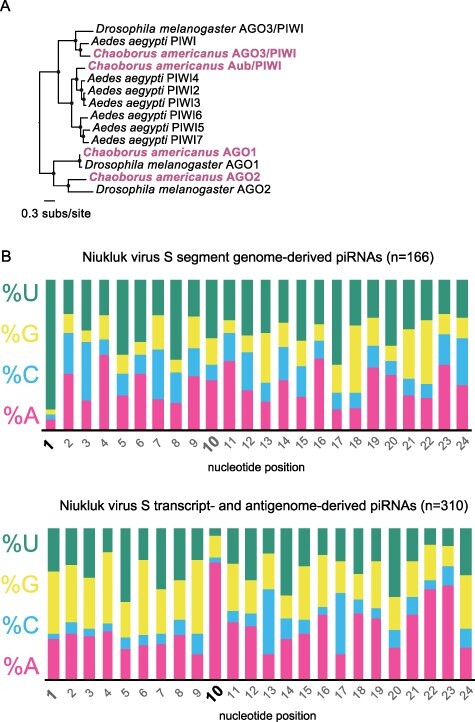
Targeting of viral RNAs by *Chaoborus* Piwi proteins. (A) Phylogenetic analysis of all Argonaute family endonucleases identified in *Chaoborus*. Nodes with FastTree maximum likelihood-like support values of 0.9 or greater are labeled with a filled circle. (B) Relative nucleotide frequencies at positions 1–24 of putative piRNAs mapped to the Niukluk virus S segment. A color legend is shown to the left of each plot. Nucleotide positions labeling the *X*-axis are emphasized for two positions at which biases are signatures of the Piwi family endonucleases Aub and Ago-3 (positions 1 and 10).

We next investigated the contribution of the piRNA pathway to the antiviral small RNA response in *Chaoborus*. In contrast to mosquitoes, phantom midges encode and express a single Argonaute 3 (AGO3)-like piwi gene and a single Aubergine-like piwi gene (Fig. 7A), suggesting a minimal role for this pathway in exogenous RNA virus infection might be expected. We assessed evidence of possible piwi contributions to virus-derived small RNA populations by combining two filtering criteria: (1) size, since piRNAs are enriched for 24–31 nt fragments, and (2) nucleotide bias, since primary piRNAs have a 5ʹ uridine (U1) bias. We focused on mapping putative piRNAs from the ovaries sample, since we expect Piwi family endonucleases to be most active in germ line tissues. While small RNAs in the 24–31 nt size range could be mapped to each viral RNA (Supplementary Table S4), an unambiguous U1 bias was only observed for those that mapped to the Niukluk virus S segment (Fig. 7B; nt frequency plots for all targets are shown in Supplementary Fig. S5).

For this viral RNA, we found that 24–31 nt small RNAs map to both strands and across the entire segment (Supplementary Fig. S6). Those derived from the negative strand show a strong U1 bias, while those derived from transcripts (or antigenomes) show a strong A10 bias (Fig. 7B). These data suggest specific targeting of the Niukluk virus S segment by *Chaoborus* piwi endonucleases. Data from additional adult tissues will be essential to further investigate the consistency and consequences of this activity. Therefore, while we find little evidence for antiviral piwi activity against many of the *Chaoborus* viruses identified here, our results are consistent with potential idiosyncratic antiviral effects arising from piRNA pathway recruitment to specific viruses or viral segments in *Chaoborus*.

### 2.8 piRNAs dominate EVE-derived small RNA populations in *Chaoborus*

**Figure 8.**
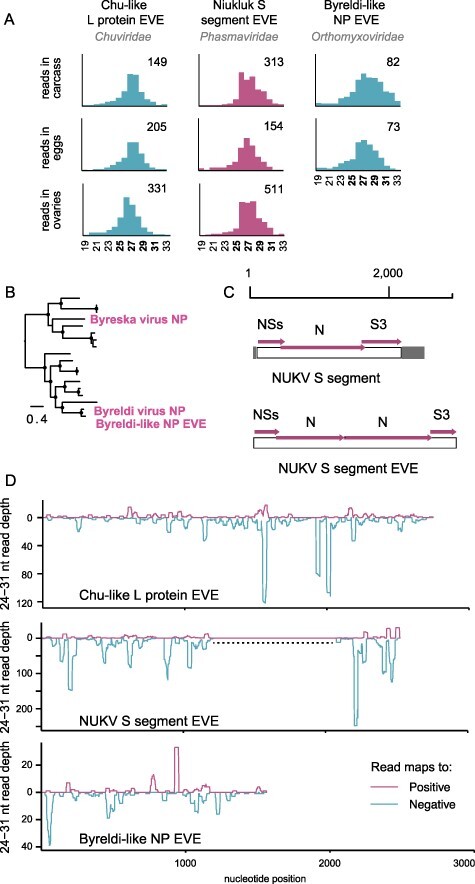
Nonretroviral EVEs in *Chaoborus*. (A) Abundant piRNAs map to novel and previously identified EVEs in the genome of *Chaoborus*. Values on the right side of each graph indicate the number of reads comprising the highest peak in each distribution. Fragment size (nt) is plotted on the *X*-axis. Alternating colors for different EVEs are used only for visual aid. (B) A phylogram of Byreska and Byreldi virus NP and the related EVE. Nodes with FastTree maximum likelihood-like support values of 0.9 or greater are labeled with a filled circle. (C) Structural diagrams of the Niukluk virus S segment and the S-derived EVE. (D) Coverage depth and strandedness of 24–31 nt reads mapping to each EVE. A dashed line indicates the duplicated region within the Niukluk virus S segment EVE.

Three contigs in the *C. americanus* RNAseq assemblies showed similarity to virus genomes but appeared incomplete or otherwise unlikely to be attributed to exogenous viruses. First, a nearly-complete NP segment similar in sequence to Byreska and Byreldi virus is present in a subset of *C. americanus* larvae from which the other five genome segments are absent. While potentially explained as a defective viral genome, small RNAs mapping to this orphan NP sequence were also detected in one of the adult small RNA datasets and are heavily enriched in the 25–30 nt size range (Fig. 8A). Phylogenetic analysis unambiguously resolves the source of this virus-like element as Byreldi virus rather than Byreska virus (Fig. 8B). Putative piRNAs were also enriched among the small RNAs mapping to a partial and pseudogenized chu-like virus L protein transcript (Fig. 8A). This sequence covers about 65 per cent of a complete L protein coding region and is 40 per cent similar to the amino acid sequences of mosquito-infecting chu-like viruses. Finally, an EVE derived from the Niukluk virus S segment, described previously (Ballinger and Taylor 2019), also yielded abundant piRNAs but no enrichment of siRNAs (Fig. 8A). For all three putative EVEs, piRNAs are abundant in both the carcasses and the ovaries or eggs, and could be amplified from *C. americanus* DNA extractions without a cDNA synthesis step. In addition, both negative- and positive-sense piRNA populations mapped from the ovaries exhibit an extreme U1 bias (Supplementary Fig. S7). Therefore, as reported for mosquitoes and other insects, nonretroviral EVEs expressed from the *Chaoborus* genome are processed into small RNAs through the piRNA pathway.

The structure of the Niukluk virus S segment EVE was previously shown to include a partial duplication of the viral nucleoprotein gene. In our present assemblies, we found the EVE covers the full coding sequence of the S segment (Fig. 8C), and like the viral RNA, piRNAs are generated from all three genes. UTRs are not identifiable in the EVE but we cannot exclude the possibility this is due to elevated substitution rates in the viral UTRs. Excluding the alignment gap created by the duplicated N region, the complete EVE and exogenous S segment share 78 per cent nucleotide identity. We considered the possibility that EVE-derived piRNAs were erroneously mapped to the exogenous Niukluk virus S segment and could explain the elevated Piwi targeting of this viral RNA, but the 22 per cent nucleotide sequence divergence between virus and EVE facilitates the unambiguous assignment of small RNAs to one or the other across most of the alignment. In the S3 coding region, there are three motifs of 31–39 consecutive nucleotides with perfect identity between the EVE and virus. Since piRNAs mapping to this region of the viral sequence cannot be confidently assigned, they were excluded from the analyses in Fig. 7B.

## 3. Discussion

In this paper, we show the stable persistence of inherited RNA viruses within parallel host lineages over evolutionary timescales. Using total and small RNA sequencing, we identified eight novel RNA viruses and confirmed their infection in larvae, adult tissues, and ovaries or eggs. Our small RNA profiling analyses implicate the siRNA pathway in host defense. EVEs derived from ancestors of two of these viruses are expressed and interact with components of the piRNA pathway, yet both viruses persist across the host species range. Further studies should examine whether EVE-derived piRNAs meaningfully contribute to controlling inherited virus replication beyond the mosquitoes (Suzuki et al. 2020), particularly in the ovaries where piRNAs are abundant and titer reductions might have the greatest impact for host fitness in the subsequent generation.

While most invertebrate viruses are known from genome sequences alone, transmission modes and fitness effects have been recently investigated for mosquito-associated lineages of several widespread invertebrate virus groups. For example, partitivirids related to Ezimos virus, Verdadero virus of *Aedes aegypti* and Galbut virus of *Drosophila melanogaster*, are inherited from both parents but are not acquired by ingestion (Cross et al. 2020). Aedes anphevirus is virtually ubiquitous in *Aedes aegypti* laboratory colonies, cell lines, and wild-caught hosts globally. Similar to the newly identified anphe-like Tolviot virus, global Aedes anphevirus strains phylogenetically group into distinct geographically-defined lineages that do not recapitulate host phylogeography (Parry and Asgari 2018). Yet, genetically diverse monophyletic anpheviruses are distributed throughout *Aedes, Anopheles*, and *Culex* species. Tolviot virus and many of the novel viruses we sequenced group immediately outside clades of mosquito-associated viruses, mirroring the evolutionary relationship of Chaoboridae and mosquitoes (Culicidae).

Our data are consistent with recent results from two other species of *Chaoborus* in which pronounced effects of Pleistocene glaciation are evident in mitochondrial genetic structure (Salmela, Härmä, and Taylor 2021). The failure of *C. americanus* to disperse among North American habitat clusters enabled the use of the insect mitochondrial clock to calibrate the timescale of virus persistence. In contrast, phylogenetic patterns consistent with frequent intraspecific sweeps and host shifts are documented throughout exemplar ISV groups, such as the sigma viruses of *Drosophila* (Carpenter et al. 2007; Longdon et al. 2011), and myriad mosquito-specific viruses (Shi et al. 2017; Parry and Asgari 2018), but whether these events represent essential strategies for long-term virus success is not known.

Unlike heritable bacterial symbionts, the more common transmission mode of inherited viruses appears to be biparental; although, our data do not demonstrate the paternal transmission of *Chaoborus* viruses. Biparental transmission can facilitate the persistence of more costly infections relative to exclusive maternal inheritance, meaning inheritance alone is not adequate evidence of a mutualistic or even commensal relationship. In the sigma viruses of *Drosophila*, negative host fitness effects are complemented by classic gene-for-gene parasite–host evolutionary dynamics in natural populations (Fleuriet and Periquet 1993; Wilfert and Jiggins 2013). In contrast, the parallel and long-term persistence of viruses in *C. americanus* without opportunity for continental sweeps suggests the fitness effects of inherited viruses vary considerably, and may fall along a parasitism-mutualism spectrum as has been proposed in other inherited microbes. However, it is also possible that the pervasive patterns of sweeps and shifts observed in other invertebrate viruses reflect opportunity rather than necessity. We view this as the less likely explanation given they coincide with the emergence and rise of host resistance. Further insight into the fitness effects of Niukluk virus and the other inherited viruses on *C. americanus* will help resolve these open questions.

Our results suggest the importance of the siRNA pathway in mediating infection by inherited viruses. We did not identify obvious differences in siRNA response between long-term members and those with regional distributions, such as Rovkytys virus and Ezimos virus, but we note the small sample size we studied limits generalization from these observations. Even so, it is striking that the only viral RNA in our dataset consistently targeted by Piwi proteins is one of the two with a corresponding EVE. The observed 22 per cent sequence divergence between Niukluk virus and its EVE in *Chaoborus* would seem to cast doubt on the possibility that these EVE piRNAs could effectively guide Piwi proteins to exogenous Niukluk virus RNA. No data are available to support whether or not perfect complementary is required for *Chaoborus* Piwi-RISC to identify targets. In *Drosophila,* only fifteen positions of perfect complementarity are required between the loaded guide RNA and target sequence for Aub-mediated cleavage (Wang et al. 2014). In an alignment of the Niukluk EVE and viral RNA, there are very few windows of at least twenty-four continuous identical positions, but there are numerous windows of 15–17 matched nucleotides.

While our data implicate neither the Niukluk S-derived EVE nor the piRNA pathway in antiviral immunity in *Chaoborus*, they suggest *Chaoborus* may be a useful system for investigating interactions between EVEs, viruses, and the piRNA pathway. Further research could help resolve new speculations raised by these results regarding the evolution of Piwi proteins as antiviral factors. For example, if it is the case that the piRNA pathway contributes to viral defense in this host, it suggests the expansion of the Piwi gene family in culicids, its apparent role in viral infection (Suzuki et al. 2020), and the association of specific gene family members with EVE-derived piRNAs (Tassetto et al. 2019) may all represent stepwise adaptive iterations from preexisting ancestral functions, in contrast to adaptations that appeared post-duplication.

Directed virome sequencing studies frequently focus on disease vectors, agricultural pests, and model organisms. *C. americanus* represents a host category that has received less attention in these surveys, but our findings emphasize these hosts are reservoirs of deeply divergent viruses. We targeted just eight larvae for virus discovery, and as a result, will have missed less prevalent members of the *C. americanus* virome. Yet the viruses we describe, including the bisegmented rhabdovirid Lantra virus, the unusually large Rovyktys virus genome, and Giez virus strains with fused and discrete coding regions, suggest these understudied corners of the invertebrate virosphere may represent environments that facilitate exploration of alternative genome architectures and are valuable targets for continued research.

### 3.1 Materials and methods

#### 3.1.1 Sample collections and tissue sources

*Chaoborus* larvae were collected from freshwater ponds by multiple oblique tows from shore with a 250 um zooplankton net. Individuals were identified to species based on diagnostic morphology in the mandibles (Uutala 1990) and preserved in cold 100 per cent ethanol for total RNA sequencing or RNAlater for small RNA sequencing. GPS coordinates of collection locations and Niukluk virus prevalence are available in Supplementary Tables S2 and S3. Live larvae were stored in 500 ml of coarse-filtered pond water and retained for rearing in the laboratory. Larvae were reared at 25°C under a 16 hr:8 hr day:night cycle and fed a zooplankton prey mix (cladocerans and copepods) from their collection location. Ovaries and eggs were sourced from unmated adult females 48 hr after eclosion. Dissections were performed with flame-sterilized forceps in sterile Ringer’s solution under a light microscope. Approximately fifty unfertilized mature oocytes comprised the majority of the visible tissue mass in the ovaries sample (Supplementary Fig. S8). In the ‘eggs’ sample, the surrounding tissue was gently removed. Both tissue collections were rinsed twice in sterile Ringer’s solution prior to homogenization. Whole third and fourth instar larvae were extracted for total RNA-Seq libraries. Whole larvae and dissected adult tissues were extracted for small RNA sequencing libraries.

#### 3.1.2 RNA extraction, library construction, and sequencing

Total RNA, including small RNAs, were extracted using the Qiagen miRNeasy kit. Larval and adult tissues were homogenized in QIAzol Lysis Reagent by 15 s of bead beating with 1-mm zirconia/silica beads (BioSpec). Before sequencing libraries were built, ribosomal RNA depletion was performed on samples for total RNA-Seq using RiboMinus reagents for eukaryotes and bacteria (Invitrogen). Barcoded RNA libraries were prepared using the NEBNext Ultra II Directional RNA Library Prep Kit (New England Biolabs). Small RNA libraries were prepared using the NEBNext Multiplex Small RNA Library Prep Set (New England Biolabs). After cDNA synthesis, small RNA libraries were size-selected from a 5 per cent polyacrylamide gel. We excised and purified fragments 140–160 bp in length, corresponding to insert sizes of 19–39 bp for siRNAs and piRNAs. Small RNAs were sequenced using 75 bp single end Illumina NextSeq sequencing at HudsonAlpha Discovery, Alabama USA. Total RNA-Seq libraries were sequenced using 150 bp paired end Illumina sequencing at Novogene Corporation, California USA.

#### 3.1.3 Sequence read trimming, assembly, and mapping

Reads were trimmed for adaptor sequences and low quality bases with BBMap 38.35 (Bushnell 2014). Metatranscriptome contigs were *de novo* assembled with Trinity 2.8.4 (Grabherr et al. 2011). Total RNA reads and small RNA reads were mapped to novel viruses using. From Utah we sequenced only small RNA libraries, so we mapped small RNAs against genomes from other regions using a more relaxed minratio parameter of 0.75. Small RNA size profiles were generated using the BBMap lhist function on mapped readsets. For coverage analyses, small RNAs were mapped by BBMap to produce BAM format read alignments. The genomecov function in BEDtools 2.29 (Quinlan and Hall 2010) was used to generate stranded mapping data and the R package ggplot2 (Wickham 2016) was used to generate plots. Detailed software parameters used in this study are present in Text S1. Positional nucleotide frequencies in putative piRNA populations were calculated in Geneious R10.

#### 3.1.4 Virus identification, sequence alignment and phylogenetics

We used a curated list of DNA and RNA virus polymerase sequences to identify novel viruses in the *C. americanus* transcriptome assemblies via tBLASTn searches. This list was built by retrieving all polymerase sequences reported in select metatranscriptomics sequencing surveys of invertebrates (Li et al. 2015; Webster et al. 2015; Shi et al. 2016) and other studies reporting highly divergent viral taxa (Obbard et al. 2020). Nucleotide sequence databases were built for each *C. americanus* metatranscriptome assembly and queried using the tblastn BLAST (Altschul et al. 1990) algorithm in Geneious R10 with an E-value cutoff of 1e^−5^. When necessary, follow-up searches were performed with appropriate search queries to identify additional genome segments. Nucleotide and amino acid alignments were performed in Geneious R10 using the MAFFT 7.388 alignment plugin set to the Auto algorithm. Phylogenetic trees were built using the FastTree 2.1.1 plugin. The GTR substitution model was used for nucleotide phylogenies and the JTT model was used for amino acid phylogenies. Accession numbers of virus sequences used as outgroups in phylogenetic analyses are present in Supplementary Table S4.

### 3.2 Host mitochondrial phylogeography

*C. americanus* COI targets were amplified by PCR and Sanger sequenced for twenty-six larvae collected in the Midwest, twenty-two from the Pacific Northwest, eighteen from the Idaho and Montana, and six from Utah. All BOLD sequences collected from the USA and Canada identified as *Chaoborus americanus* or *Chaoborus sp.* were retrieved in March 2021. Nucleotide sequences were aligned in Geneious R10 using the MAFFT 7.388 plugin set to the Auto algorithm and FastTree 2.1.1 was used to build a phylogeny under the GTR substitution model. The BOLD list was manually curated in two ways, (1) to remove *Chaoborus sp.* entries that grouped outside the monophyletic *C. americanus* clade recovered, with *Chaoborus flavicans* used as the outgroup, and (2) to reduce sequence redundancy when three or more identical sequences were present from a single collection location.

#### 3.2.1 Virus screening and Sanger sequencing

Niukluk virus screens were performed on aliquots of total RNA extractions from each of 353 *C. americanus* (Supplementary Tables S2 and S3). cDNA synthesis for screens was done using Superscript III Reverse Transcriptase (Invitrogen) and primed with random hexamers (IDT). Primers used for PCR are listed in Supplementary Table S5. A touchdown program was used for all PCR reactions and consisted of an initial denaturation step of 95°C for 120 s, followed by 10 cycles of 30 s at 95°C, 30 s at 54°C (−0.6°C/cycle), and 60 s at 72°C, then 30 cycles of 30 s at 95°C, 30 s at 48°C, and 60 s at 72°C. For reactions generating long Niukluk virus fragments for Sanger sequencing the 60 s extension was increased to 120 s. Recombinant Taq DNA polymerase and PCR reaction buffer were sourced from ThermoFisher (EP0402) and dNTPs from Invitrogen (10297117). All custom primers used in this study were synthesized by IDT. Sanger sequencing was used to supplement Niukluk virus genomes acquired through RNA-Seq and focused on increasing sampling in British Columbia and Wisconsin. Amplicons were sequenced for the L (∼900 bp), M (∼1,400 bp), S (∼950 bp), and host mitochondrial COI (∼730 bp).

#### 3.2.2 Virus names

All novel viruses in this study were assigned names derived from the High Valyrian language from the fantasy series, *Game of Thrones*.

## Supplementary Material

veac018_Supp

## Data Availability

All sequence data generated and analyzed during this work can be accessed under NCBI BioProject PRJNA737432. Additional supplemental data including assembled virus genome sequences, alignments used for phylogenetic analyses, and mapping data can be accessed at 10.6084/m9.figshare.c.5858478.
