# Supplementary material for: Evolution and diversity of inherited viruses in the Nearctic phantom midge, Chaoborus americanus
Source: Virus Evol. 2022 Mar 10;8(1):veac018. doi: 10.1093/ve/veac018 (PMC8963322; doi:10.1093/ve/veac018)
Supplement: veac018_Supp [file veac018_supp.zip › Supplementary Tables and Figures.pdf]

Table S1. *Chaoborus americanus* sequencing libraries generated for this study.

Sample	Location	Tissue	Library type	Total reads
SNO1 (2019)	Snoqualmie, Washington, USA	Larva	Total RNA	111185152
SNO2 (2019)	Snoqualmie, Washington, USA	Larva	Total RNA	107982356
SNO3	Snoqualmie, Washington, USA	Larva	Total RNA	147970646
BW1	Garibaldi, BC, Canada	Larva	Total RNA	111784330
BW2	Garibaldi, BC, Canada	Larva	Total RNA	173119484
BW5	Garibaldi, BC, Canada	Larva	Total RNA	123562542
IRN3	Iron River, Wisconsin, USA	Larva	Total RNA	109090290
IRN4	Iron River, Wisconsin, USA	Larva	Total RNA	131969984
SNOA1C	Snoqualmie, Washington, USA	Adult carcass	Small RNA	20155818
SNOA1E	Snoqualmie, Washington, USA	Eggs	Small RNA	21279218
SNOA3C	Snoqualmie, Washington, USA	Adult carcass	Small RNA	20253182
SNOA3O	Snoqualmie, Washington, USA	Eggs, ovaries	Small RNA	18856286
SNO1 (2020)	Snoqualmie, Washington, USA	Larva	Small RNA	25415769
SNO2 (2020)	Snoqualmie, Washington, USA	Larva	Small RNA	24755932
SNO5	Snoqualmie, Washington, USA	Larva	Small RNA	26303767
SNO7	Snoqualmie, Washington, USA	Larva	Small RNA	22463074
SNO11	Snoqualmie, Washington, USA	Larva	Small RNA	25726035
SNO15	Snoqualmie, Washington, USA	Larva	Small RNA	24047982
TEA1	Delta, Wisconsin, USA	Larva	Small RNA	26050755
TEA5	Delta, Wisconsin, USA	Larva	Small RNA	24713693
TEA6	Delta, Wisconsin, USA	Larva	Small RNA	24906827
TEA7	Delta, Wisconsin, USA	Larva	Small RNA	25103523
TEA10	Delta, Wisconsin, USA	Larva	Small RNA	24987476
TEA12	Delta, Wisconsin, USA	Larva	Small RNA	25003313
TEA15	Delta, Wisconsin, USA	Larva	Small RNA	23806282
TEA18	Delta, Wisconsin, USA	Larva	Small RNA	20967161
UIN1	Uinta-Wasatch-Cache, Utah, USA	Larva	Small RNA	18225673
UIN3	Uinta-Wasatch-Cache, Utah, USA	Larva	Small RNA	20379594

Table S2. Niukluk phantom virus infection prevalence by region.

Region	Ponds sampled	*C. americanus* sites	Indv. screened	Inf. freq. (%)
WA, BC	26	4	111	24.3
ID, MT	9	2	35	0.0
UT	7	2	49	2.0
ND, MN, WI, MI	17	8	158	7.0
Total	59	16	353	11.1
AK*	345	7†	116	61.0

*previous long term zooplankton survey study

†indicates *C. americanus* sites screened for NUKV

Table S3. Niukluk phantom virus prevalence by population.

Location	ID	Screened	Infected	Inf. freq. (%)	GPS (North)	GPS (West)
Snoqualmie, WA 2019	SNO_19	18	8	44.4	47°25'2.16"	121°24'29.63"
Snoqualmie, WA 2020	SNO_20	23	12	52.2	47°25'2.16"	121°24'29.63"
Garibaldi, BC	BW	30	7	23.3	50° 2'23.95"	123° 7'10.33"
Shelton, WA	EFC	20	0	0.0	47°17'33.12"	123° 6'54.64"
Forlorn Lakes, WA	FOR	20	0	0.0	45°57'47.42"	121°44'19.85"
Kellogg, ID	KEL	16	0	0.0	47°32'19.88"	116° 8'33.17"
Frenchtown, MT	FRE	19	0	0.0	47° 1'44.82"	114°17'43.70"
Uinta 1, UT	UIN1	29	1	3.4	40°43'6.12"	110°52'7.07"
Uinta 2, UT	UIN2	20	0	0.0	40°40'56.94"	110°57'54.25"
Lake Park, MN	LAK	19	0	0.0	46°53'5.36"	96° 4'38.13"
Eglon, MN	EGL	16	0	0.0	46°52'35.06"	96°10'52.54"
Smoky Hills, MN	SMK	8	0	0.0	46°54'31.79"	95°20'7.55"
Iron River, WI 2019	IRN1_19	28	2	7.1	46°33'48.90"	91°20'34.51"
Iron River, WI 2020	IRN1_20	30	0	0.0	46°33'48.90"	91°20'34.51"
Iron River, WI 2020	IRN2	20	0	0.0	46°33'36.51"	91°20'17.37"
Delta, WI	TEA	20	9	45.0	46°25'49.74"	91°15'46.34"
Munising, MI	MUN	16	0	0.0	46°23'5.04"	86°40'11.42"

Table S4. Sequence names and accession numbers used for virus phylogenetic analysis in this paper.

Virus family; gene	Sequence description	Organism	NCBI accession
Orthomyxoviridae; PB1	PB1	[Aedes alboannulatus orthomyxo-like virus]	ASA47420.1
Orthomyxoviridae; PB1	PB1	[Guadeloupe mosquito quaranja-like virus 3]	QEM39320.1
Orthomyxoviridae; PB1	PB1	[Jingshan Fly Virus 1]	AJG39084.1
Orthomyxoviridae; PB1	PB1	[Splett orthomyxo-like virus]	QIJ70033.1
Orthomyxoviridae; PB1	PB1	[Whidbey virus]	AQU42764.1
Orthomyxoviridae; PB1	PB1	[Wuhan Louse Fly Virus 3]	AJG39089.1
Orthomyxoviridae; PB1	PB1	[Wuhan Louse Fly Virus 4]	AJG39090.1
Orthomyxoviridae; PB1	PB1	[Wuhan Mosquito Virus 3]	AJG39091.1
Orthomyxoviridae; PB1	PB1	[Wuhan Mosquito Virus 4]	AJG39092.1
Orthomyxoviridae; PB1	PB1	[Wuhan Mosquito Virus 5]	AJG39093.1
Orthomyxoviridae; PB1	PB1 polymerase	[Aedes detritus orthomyxo-like virus]	QRD99912.1
Orthomyxoviridae; PB1	PB1 polymerase	[Culex modestus orthomyxo-like virus]	QRD99916.1
Orthomyxoviridae; PB1	PB1 polymerase	[Culex pipiens orthomyxo-like virus]	QRD99919.1
Orthomyxoviridae; PB1	polymerase PB1	[Dipteran orthomyxo-related virus OKIAV193]	QMP82272.1
Orthomyxoviridae; PB1	polymerase PB1	[Hemipteran orthomyxo-related virus OKIAV188]	QMP82403.1
Orthomyxoviridae; PB1	polymerase PB1	[Hemipteran orthomyxo-related virus OKIAV191]	QMP82202.1
Orthomyxoviridae; PB1	polymerase PB1	[Neuropteran orthomyxo-related virus OKIAV190]	QPL15288.1
Orthomyxoviridae; PB1	polymerase PB1	[Astopletus virus]	QRW42569.1
Orthomyxoviridae; PB1	polymerase PB1	[Culex orthomyxo-like virus]	QGA87318.1
Orthomyxoviridae; PB1	polymerase PB1	[Dipteran orthomyxo-related virus OKIAV195]	QMP82229.1
Orthomyxoviridae; PB1	polymerase PB1	[Guadeloupe mosquito quaranja-like virus 1]	QRW42591.1
Orthomyxoviridae; PB1	polymerase PB1	[Usinis virus]	QRW42655.1
Orthomyxoviridae; PB1	polymerase PB1	[Wuhan Mosquito Virus 6]	QRW42454.1
Orthomyxoviridae; PB2	MAG polymerase PB2	[Orthomyxoviridae sp.]	QYW05798.1
Orthomyxoviridae; PB2	MAG polymerase PB2	[Orthomyxoviridae sp.]	QYW05796.1
Orthomyxoviridae; PB2	PB2	[Guadeloupe mosquito quaranja-like virus 3]	QEM39323.1
Orthomyxoviridae; PB2	PB2	[Whidbey virus]	AQU42765.1
Orthomyxoviridae; PB2	PB2	[Aedes alboannulatus orthomyxo-like virus]	ASA47421.1
Orthomyxoviridae; PB2	PB2	[Old quarry swamp virus]	AYP67574.1
Orthomyxoviridae; PB2	PB2 polymerase	[Aedes detritus orthomyxo-like virus]	QRD99913.1
Orthomyxoviridae; PB2	PB2 polymerase	[Culex modestus orthomyxo-like virus]	QRD99917.1
Orthomyxoviridae; PB2	PB2 polymerase	[Culex pipiens orthomyxo-like virus]	QRD99918.1
Orthomyxoviridae; PB2	polymerase PB2	[Dipteran orthomyxo-related virus OKIAV193]	QMP82135.1
Orthomyxoviridae; PB2	polymerase PB2	[Guadeloupe mosquito quaranja-like virus 1]	QRW42586.1
Orthomyxoviridae; PB2	polymerase PB2	[Neuropteran orthomyxo-related virus OKIAV190]	QPL15367.1
Orthomyxoviridae; PB2	polymerase PB2	[Astopletus virus]	QRW42567.1
Orthomyxoviridae; PB2	polymerase PB2	[Culex orthomyxo-like virus]	QGA87319.1
Orthomyxoviridae; PB2	polymerase PB2	[Jingshan Fly Virus 1]	APG77877.1
Orthomyxoviridae; PB2	polymerase PB2	[Sanxia Water Strider Virus 3]	APG77901.1
Orthomyxoviridae; PB2	polymerase PB2	[Usinis virus]	QRW42644.1
Orthomyxoviridae; PB2	polymerase PB2	[Wuhan Mosquito Virus 4]	APG77861.1
Orthomyxoviridae; PB2	polymerase PB2	[Wuhan Mosquito Virus 6]	QRW42439.1
Partitiviridae; RdRp	hypothetical protein	[Leuven Partiti-like virus 3]	QZZ63397.1
Partitiviridae; RdRp	MAG RNA-dependent RNA polymerase	[Riboviria sp.]	UCS96350.1
Partitiviridae; RdRp	MAG RNA-dependent RNA polymerase	[Riboviria sp.]	UCS96396.1
Partitiviridae; RdRp	RdRp	[Hubei partiti-like virus 8]	APG78307.1
Partitiviridae; RdRp	RdRp	[Hubei partiti-like virus 15]	APG78282.1
Partitiviridae; RdRp	RdRp	[Partitivirus-like 2]	AOR51389.1
Partitiviridae; RdRp	RdRp	[Beihai barnacle virus 12]	YP_009333370.1
Partitiviridae; RdRp	RdRp	[Beihai partiti-like virus 2]	YP_009333350.1
Partitiviridae; RdRp	RdRp	[Hubei diptera virus 17]	YP_009337870.1
Partitiviridae; RdRp	RdRp	[Hubei partiti-like virus 4]	APG78224.1
Partitiviridae; RdRp	RdRp	[Hubei partiti-like virus 5]	APG78252.1
Partitiviridae; RdRp	RdRp	[Hubei partiti-like virus 9]	APG78308.1

Partitiviridae; RdRp	RdRp	[Hubei partiti-like virus 10]	APG78227.1
Partitiviridae; RdRp	RdRp	[Hubei partiti-like virus 11]	YP_009329875.1
Partitiviridae; RdRp	RdRp	[Hubei partiti-like virus 13]	APG78275.1
Partitiviridae; RdRp	RdRp	[Hubei partiti-like virus 14]	APG78278.1
Partitiviridae; RdRp	RdRp	[Hubei partiti-like virus 16]	APG78316.1
Partitiviridae; RdRp	RdRp	[Partitivirus-like 3]	AOR51390.1
Partitiviridae; RdRp	RdRp	[Wuhan cricket virus 2]	YP_009345133.1
Partitiviridae; RdRp	RNA-dependent RNA polymerase	[Riboviria sp.]	QIM73956.1
Partitiviridae; RdRp	RNA-dependent RNA polymerase	[Soufli partiti-like virus]	QRD99865.1
Partitiviridae; RdRp	RNA-dependent RNA polymerase	[Dragana partiti-like virus]	QRD99907.1
Partitiviridae; RdRp	RNA-dependent RNA polymerase	[Partitivirus-like Culex mosquito virus]	AXQ04877.1
Partitiviridae; RdRp	RNA-dependent RNA polymerase	[Sonnbo virus]	QGA70940.1
Partitiviridae; RdRp	RNA-dependent RNA polymerase	[Verdadero virus]	QMI58128.1
Partitiviridae; Chaq-like ORF	hypothetical protein	[Chaq virus]	AWY11087.1
Partitiviridae; Chaq-like ORF	hypothetical protein	[Chaq virus]	AWY11113.1
Partitiviridae; Chaq-like ORF	hypothetical protein	[Chaq virus]	AWY11175.1
Partitiviridae; Chaq-like ORF	hypothetical protein	[Didymoteicho chaq virus]	QRD99871.1
Partitiviridae; Chaq-like ORF	hypothetical protein	[Nefer virus]	QRW42512.1
Partitiviridae; Chaq-like ORF	orf1	[Chaq virus]	AKH40308.1
Partitiviridae; Chaq-like ORF	ORF1	[Chaq virus-like 1]	AOR51384.1
Partitiviridae; Chaq-like ORF	ORF1	[Chaq virus-like 2]	AOR51385.1
Partitiviridae; Chaq-like ORF	ORF1	[Chaq virus-like 3]	AOR51386.1
Partitiviridae; Chaq-like ORF	ORF	[Chaq-like virus]	QMI58130.1
Partitiviridae; Chaq-like ORF	ORF	[Chaq virus]	QMI58117.1
Phasmaviridae; L protein	RNA-dependent RNA polymerase	Anopheles triannulatus orthophasmavirus	MH822966
Phasmaviridae; L protein	RNA-dependent RNA polymerase	Barstukas virus	MW434660
Phasmaviridae; L protein	RNA-dependent RNA polymerase	Coleopteran phasma-related virus	MT153542
Phasmaviridae; L protein	RNA-dependent RNA polymerase	Coredo virus	MN661021
Phasmaviridae; L protein	RNA-dependent RNA polymerase	Culex phasma-like virus	MF176242
Phasmaviridae; L protein	RNA-dependent RNA polymerase	Flen virus	MN513376
Phasmaviridae; L protein	RNA-dependent RNA polymerase	Ganda bee virus	KY053854
Phasmaviridae; L protein	RNA-dependent RNA polymerase	Hubei odonate virus 8	KX884775
Phasmaviridae; L protein	RNA-dependent RNA polymerase	Hubei odonate virus 9	KX884786
Phasmaviridae; L protein	RNA-dependent RNA polymerase	TSA: Chrysis fasciata (Hymenoptera phasma-like virus)	GBMK01013408
Phasmaviridae; L protein	RNA-dependent RNA polymerase	TSA: Philoctetes bogdanovii (Hymenoptera phasma-like virus)	GBVA01004800
Phasmaviridae; L protein	RNA-dependent RNA polymerase	Kigluaik phantom virus	KJ434182
Phasmaviridae; L protein	RNA-dependent RNA polymerase	Miglotas virus	MW434703
Phasmaviridae; L protein	RNA-dependent RNA polymerase	Niukluk phantom virus	MN168168
Phasmaviridae; L protein	RNA-dependent RNA polymerase	Scaphoideus titanus bunya-like virus 1	MN982386
Phasmaviridae; L protein	RNA-dependent RNA polymerase	Wuchang cockroach virus 1	KM817688
Phasmaviridae; L protein	RNA-dependent RNA polymerase	Wuhan mosquito virus 1	KM817697
Phasmaviridae; L protein	RNA-dependent RNA polymerase	Wuhan mosquito virus 2	KM817698
Totiviridae; RdRp	hypothetical protein 2	[Hubei toti-like virus 10]	YP_009336493.1
Totiviridae; RdRp	hypothetical protein	[Hubei toti-like virus 7]	APG76025.1
Totiviridae; RdRp	PArp-RdRp	[dsRNA virus environmental sample]	AJT39583.1
Totiviridae; RdRp	polymerase	[Schistocephalus solidus toti-like virus 2]	QJD26158.1
Totiviridae; RdRp	putative RNA dependent RNA polymerase	[Thrips tabaci associated dsRNA virus 1]	QNS31034.1
Totiviridae; RdRp	putative RNA dependent RNA polymerase	[Thrips tabaci associated dsRNA virus 2]	QNS31036.1
Totiviridae; RdRp	putative RNA-dependent RNA polymerase	[uncultured virus]	AGW51771.1
Totiviridae; RdRp	RdRp	[Aedes alboannulatus toti-like virus 1]	YP_009388609.1
Totiviridae; RdRp	RdRp	[Aedes camptorhynchus toti-like virus 1]	YP_009388611.1
Totiviridae; RdRp	RdRp	[Atrato virus]	QHA33708.1
Totiviridae; RdRp	RdRp	[Murri virus]	QHA33714.1
Totiviridae; RdRp	RNA-dependent RNA polymerase	[Persimmon latent virus]	YP_009025166.1
Totiviridae; RdRp	RNA-dependent RNA polymerase	[Aedes aegypti toti-like virus]	QEM39133.1
Totiviridae; RdRp	RNA-dependent RNA polymerase	[Culex vishnui subgroup totivirus]	BBQ05098.1

Totiviridae; RdRp	RNA-dependent RNA polymerase	[Gouley virus]	QRW41694.1
Totiviridae; RdRp	RNA-dependent RNA polymerase	[Lotchka virus]	QRW42121.1
Totiviridae; RdRp	RNA-dependent RNA polymerase	[Mika virus]	QRW41697.1
Totiviridae; RdRp	RNA-dependent RNA polymerase	[Snelk virus]	QRW41776.1
Totiviridae; RdRp	RNA-dependent RNA polymerase	[Stinn virus]	QRW41701.1
Totiviridae; RdRp	RNA-dependent RNA polymerase	[Totiviridae sp.]	QKN88741.1
Totiviridae; RdRp	RNA-dependent RNA polymerase	[Tzifr virus]	QRW42115.1
Totiviridae; RdRp	RNA-directed RNA polymerase	[Circulifer tenellus virus 1]	YP_003800003.1
Totiviridae; RdRp	RNA-directed RNA polymerase	[Scaphoideus titanus toti-like virus 1]	QIJ56903.1
Totiviridae; RdRp	RNA-directed RNA polymerase	[Spissistilus festinus virus 1]	YP_003800001.1
Mononegavirales; RdRp	large polymerase protein	[Isfahan virus]	YP_007641386.1
Mononegavirales; RdRp	large protein	[Riverside virus 1]	AMJ52368.1
Mononegavirales; RdRp	L polymerase protein	[Vesicular stomatitis New Jersey virus]	ASR83122.1
Mononegavirales; RdRp	L protein	[Berant virus]	AOC55081
Mononegavirales; RdRp	MAG RNA-dependent RNA polymerase	[Canya virus]	QRW41710
Mononegavirales; RdRp	polymerase	[Jurona vesiculovirus]	YP_009513007.1
Mononegavirales; RdRp	polymerase	[Morreton vesiculovirus]	YP_009362086.1
Mononegavirales; RdRp	polymerase-associated protein	[Ohlsdorf virus]	ATG83562.1
Mononegavirales; RdRp	polymerase protein	[Vesicular stomatitis Indiana virus]	AYK03298.1
Mononegavirales; RdRp	putative polyprotein	[Lampyris noctiluca rhabdo-like virus 1]	QBP37026
Mononegavirales; RdRp	putative RdRp-complex	[Linepithema humile rhabdo-like virus 1]	AXA52562
Mononegavirales; RdRp	putative replicase	[Jimsystermes virus]	QQM16270
Mononegavirales; RdRp	putative RNA-dependent RNA polymerase	[Culex rhabdo-like virus]	AXQ04772.1
Mononegavirales; RdRp	putative RNA-dependent RNA polymerase	[Tongilchon virus 1]	YP_009182186.1
Mononegavirales; RdRp	RdRp	[Atrato Rhabdo-like virus 2]	QHA33839
Mononegavirales; RdRp	RdRP	[Anopheles darlingi virus]	QBK47204.1
Mononegavirales; RdRp	RdRP	[Anopheles marajoara virus]	QBK47216.1
Mononegavirales; RdRp	RdRp	[Bolahun virus variant 1]	AOR51366.1
Mononegavirales; RdRp	RdRp	[Culex mononega-like virus 1]	ASA47403.1
Mononegavirales; RdRp	RdRp	[Culex mononega-like virus 2]	ASA47322.1
Mononegavirales; RdRp	RdRp	[Culex rhabdo-like virus]	ASA47473.1
Mononegavirales; RdRp	RdRp	[Gambie virus]	AOR51378.1
Mononegavirales; RdRp	RdRp	[Grenada mosquito rhabdovirus 1]	AVP26977
Mononegavirales; RdRp	RdRP protein	[Aedes anphevirus]	AWW13453.1
Mononegavirales; RdRp	RNA-depedent RNA polymerase	[Maraba virus]	YP_009091830.1
Mononegavirales; RdRp	RNA-dependent RNA polymerase	[Blattodean arli-related virus OKIAV101]	QMP82176
Mononegavirales; RdRp	RNA-dependent RNA polymerase	[Hemipteran rhabdo-related virus OKIAV26]	QMP82309
Mononegavirales; RdRp	RNA-dependent RNA polymerase	[Hymenopteran arli-related virus OKIAV100]	QPL15365
Mononegavirales; RdRp	RNA-dependent RNA polymerase	[Jingshan Fly Virus 2]	AJG39108
Mononegavirales; RdRp	RNA-dependent RNA polymerase	[Megalopteran arli-related virus OKIAV106]	QMP82230
Mononegavirales; RdRp	RNA-dependent RNA polymerase	[Neuropteran arli-related virus OKIAV105]	QPL15298
Mononegavirales; RdRp	RNA-dependent RNA polymerase	[Aedes aegypti anphevirus]	AWK27462.1
Mononegavirales; RdRp	RNA-dependent RNA polymerase	[Aedes albopictus anphevirus]	QOW17628.1
Mononegavirales; RdRp	RNA-dependent RNA polymerase	[Amsterdam virus]	QEQ50497
Mononegavirales; RdRp	RNA-dependent RNA polymerase	[Blattodean arli-related virus OKIAV102]	QMP82242
Mononegavirales; RdRp	RNA-dependent RNA polymerase	[Carp sprivivirus]	AXA12029.1
Mononegavirales; RdRp	RNA-dependent RNA polymerase	[Coleopteran arli-related virus OKIAV107]	QMP82314
Mononegavirales; RdRp	RNA-dependent RNA polymerase	[Coleopteran rhabdo-related virus OKIAV28]	QMP82194
Mononegavirales; RdRp	RNA-dependent RNA polymerase	[Coleopteran rhabdo-related virus OKIAV29]	QMP82342
Mononegavirales; RdRp	RNA-dependent RNA polymerase	[Culex pseudovishnui rhabdo-like virus]	BBQ04827.1
Mononegavirales; RdRp	RNA-dependent RNA polymerase	[Culex tritaeniorhynchus Anphevirus]	BBQ04822.1
Mononegavirales; RdRp	RNA-dependent RNA polymerase	[Drosophila busckii rhabdovirus]	AMK09240
Mononegavirales; RdRp	RNA-dependent RNA polymerase	[Elisy virus]	QRW41819
Mononegavirales; RdRp	RNA-dependent RNA polymerase	[Gordis virus]	QRW42745.1
Mononegavirales; RdRp	RNA-dependent RNA polymerase	[Guadeloupe Culex rhabdovirus]	QEM39085
Mononegavirales; RdRp	RNA-dependent RNA polymerase	[Guadeloupe mosquito mononega-like virus]	QEM39177.1

Mononegavirales; RdRp	RNA-dependent RNA polymerase	[Hubei rhabdo-like virus 3]	YP_009336889
Mononegavirales; RdRp	RNA-dependent RNA polymerase	[Hubei rhabdo-like virus 7]	YP_009337121.1
Mononegavirales; RdRp	RNA-dependent RNA polymerase	[Hymenopteran arli-related virus OKIAV98]	QPL15295
Mononegavirales; RdRp	RNA-dependent RNA polymerase	[Hymenopteran arli-related virus OKIAV99]	QPL15345
Mononegavirales; RdRp	RNA-dependent RNA polymerase	[Isopteran arli-related virus OKIAV103]	QMP82349
Mononegavirales; RdRp	RNA-dependent RNA-polymerase	[Malpais Spring vesiculovirus]	YP_009094178.1
Mononegavirales; RdRp	RNA-dependent RNA polymerase	[Mononegavirales sp.]	QTW97812.1
Mononegavirales; RdRp	RNA dependent RNA polymerase	[North Creek virus]	AGY80343.1
Mononegavirales; RdRp	RNA-dependent RNA polymerase	[Odonatan anphe-related virus OKIAV59]	QMP82151.1
Mononegavirales; RdRp	RNA-dependent RNA polymerase	[Piry virus]	YP_009505536.1
Mononegavirales; RdRp	RNA-dependent RNA polymerase	[Sanxia Water Strider Virus 5]	YP_009289352
Mononegavirales; RdRp	RNA-dependent RNA polymerase	[Serbia mononega-like virus 1]	QNS17450.1
Mononegavirales; RdRp	RNA-dependent RNA polymerase	[Stang virus]	QRW41829
Mononegavirales; RdRp	RNA-dependent RNA polymerase	[Strepsipteran arli-related virus OKIAV104]	QMP82292
Mononegavirales; RdRp	RNA-dependent RNA polymerase	[Tacheng Tick Virus 6]	YP_009304420
Mononegavirales; RdRp	RNA-dependent RNA polymerase	[Wuchang romanomermis nematode virus 2]	YP_009342285
Mononegavirales; RdRp	RNA-dependent RNA polymerase	[Wuhan Mosquito Virus 9]	QTW97825
Mononegavirales; RdRp	RNA-dependent RNA polymerase	[Xincheng Mosquito Virus]	YP_009302387.1
Mononegavirales; RdRp	RNA-directed RNA polymerase L	[Papilio machaon]	KPJ12187.1
Mononegavirales; RdRp	RNA polymerase	[Grass carp virus]	YP_009094267.1
Mononegavirales; RdRp	RNA polymerase	[Tench rhabdovirus]	YP_009094206.1
Mononegavirales; RdRp	polyprotein-like gene	[UNVERIFIED: Lobeira virus isolate BR/MT_M05]	MK780203

Table S5. PCR primers used in this study.

Target	Primer 1	Primer 2	Application
Niukluk virus L segment	GTGGTGGCAGACCTATATCGACTCC	CTAATTCACCTAGATATTTACTACCCCA	Virus screening
Niukluk virus M segment	ATTCAAGGGGTTGCATTTTGGATT	GTGCTTGTTATGTATAATGTGTTATCACA	Virus screening
Niukluk virus S segment	ACTCTGATTCCCATAGGACAAAGGTCGT	TACAGTCATATCTCTGAGTGTGCCATCTG	Virus screening
Niukluk virus L segment	TGGAAGTGTTCATACACCTATTTCAAAGAC	ATTATCAGCACAAAATGCTGTCTCC	Long product for Sanger sequencing
Niukluk virus M segment	AAGATGRTATGTAARCARTGCCTATT	CTAAGCAAGAGTAAGTTGTCATTAC	Long product for Sanger sequencing
Niukluk virus S segment	GAGTGTCCAGCAAGCACTG	TGATGCTTCTCCTGATTCATTGGA	Long product for Sanger sequencing
Chaoborus americanus COI	GCGACAATGATTATTTTCGACAAATC	AATATGGGAGATTATTCCAAAGCCAGG	Sanger sequencing
Lantra virus RNA2 (L) Primer A (Figure 4C)	CTGGCTCATTCTCAAGCTACAGA	-	Lantra virus genome segmentation test
Lantra virus RNA2 (L) Primer B (Figure 4C)	GACTGTTGGTCTCGTATGGAGTTATC	-	Lantra virus genome segmentation test
Lantra virus RNA2 (L) Primer C (Figure 4C)	CTCCTATATCTTCCTCAAAATTGTCATCC	-	Lantra virus genome segmentation test
Lantra virus RNA1 (NPMG) Primer D (Figure 4C)	TTTATTCCCTTTCTCCTGCTGGTC	-	Lantra virus genome segmentation test
Lantra virus RNA1 (NPMG) Primer E (Figure 4C)	CTGATATTGACTGCACTTAACAGTGAG	-	Lantra virus genome segmentation test
Lantra virus RNA1 (NPMG) Primer F (Figure 4C)	GGAGAGAATGAGGAAATCTGATCCT	-	Lantra virus genome segmentation test
Lantra virus RNA1 (NPMG) Primer G (Figure 4C)	AGTGAACCGATCCATACAGGC	-	Lantra virus genome segmentation test
Niukluk S segment EVE	AACATAGAACAAAGGTTAGTGAGTGT	ATAGCATTTATTATGTCTGTCCGAGT	EVE DNA amplification
Chu-like RdRp EVE	TGCACAAGGTGTTCTTAGGTCAT	ATGGGAGCTTAATGAAGTCGCTT	EVE DNA amplification
Byreldi virus NP EVE	AGAGCCACGCCTAACATAAATGT	ATCCTGTTGCTTCCCACTTCATT	EVE DNA amplification

Figure S1. Phylogeographic structure of *Chaoborus americanus* in North America. Phylogenetic relationships of the cytochrome oxidase subunit I (COI) barcode region. All *C. americanus* sequences in the Barcode of Life Database are included alongside representative of all regions sampled in the current study. Tips are color-coded to sampling locations. Branches are labeled with FastTree support values greater than 0.75. The tree is rooted on the *Chaoborus flavicans* and *Chaoborus* sp. sequences, and branches leading to these taxa are artificially truncated.

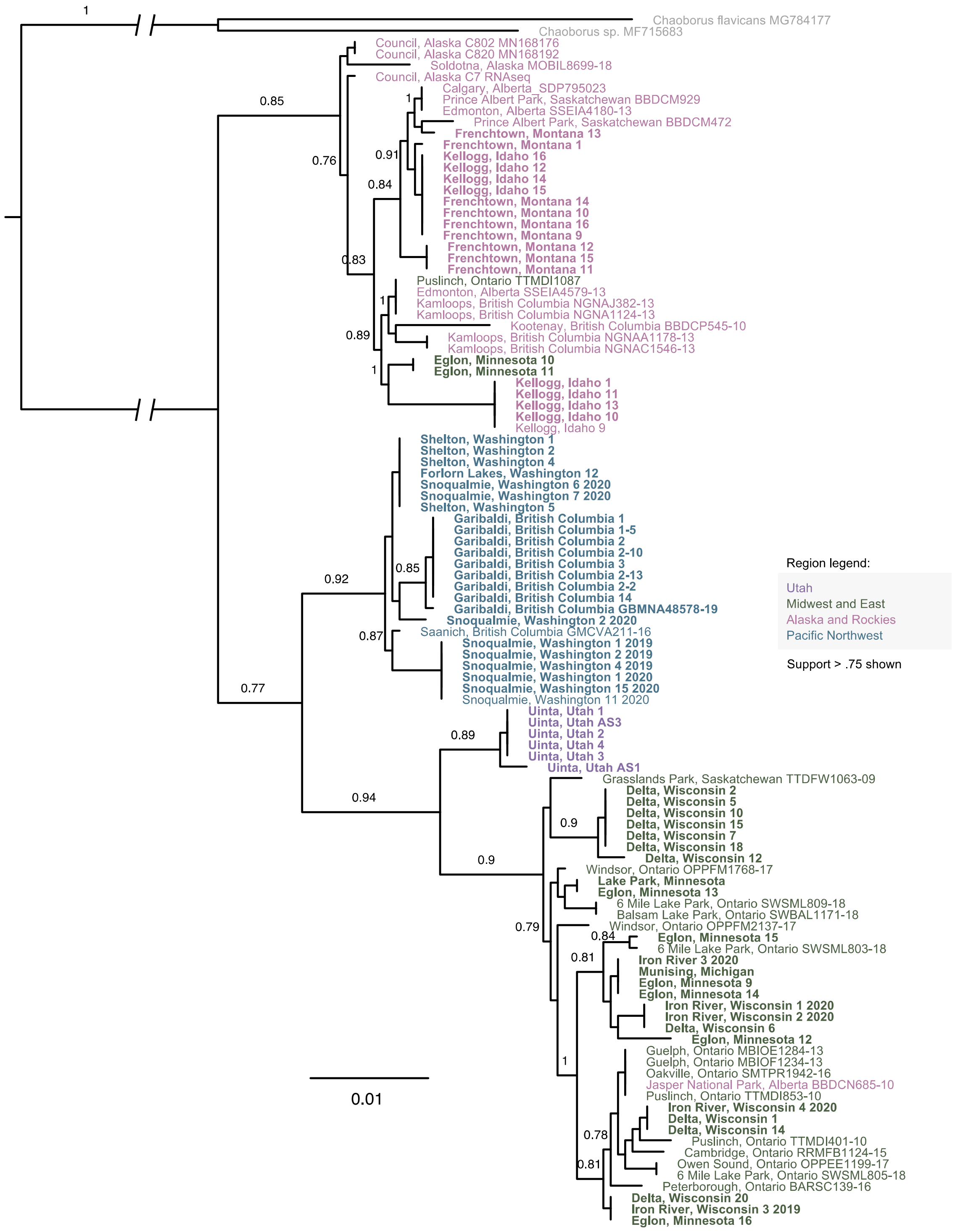

Figure S2. Small RNA profiles of an orthomyxovirid and related EVE in larval *C. americanus* collected in Wisconsin. A) Small RNAs mapped against each of the six putative segments of Byreska virus exhibit small RNA size profiles consistent with their processing by the siRNA pathway endonuclease Dicer-2. B) The Byreldi virus-like NP sequence, which recovered piRNA-dominated small RNA profiles and amplified from DNA templates in adults from Washington (right) recover a similar piRNA-like profile in larvae from Wisconsin. Virus plots are colored magenta and EVE plots are colored teal.

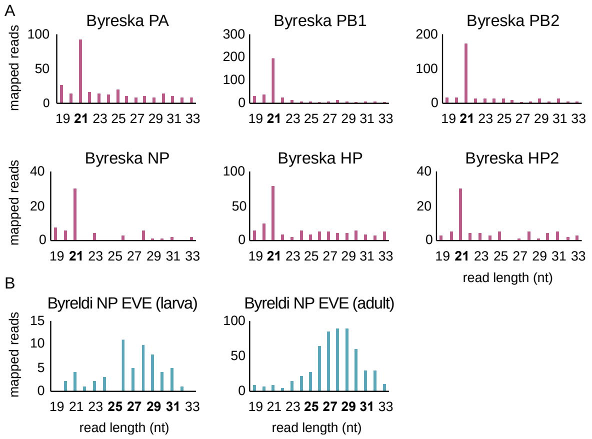

(next page) Figure S3. Spatial distribution and abundance of small interfering RNAs derived from *Chaoborus* viruses. A) Distribution of 21 nt small RNAs mapped to *Chaoborus americanus* viruses. Graph are labeled with virus (and segment) names. All viruses except Byreska were mapped to reads sequenced from adult tissue. Byreska virus reads were mapped from an infected larva. Plot color and orientation corresponds to reads mapping to the positive strand (upper plots) or negative strand (lower plots) in each graph. Genome position, on the X axis, references the negative sense genome, except for Giez virus and Ezimos virus, which are dsRNA. For these viruses, reads were mapped to a positive sense reference sequence.

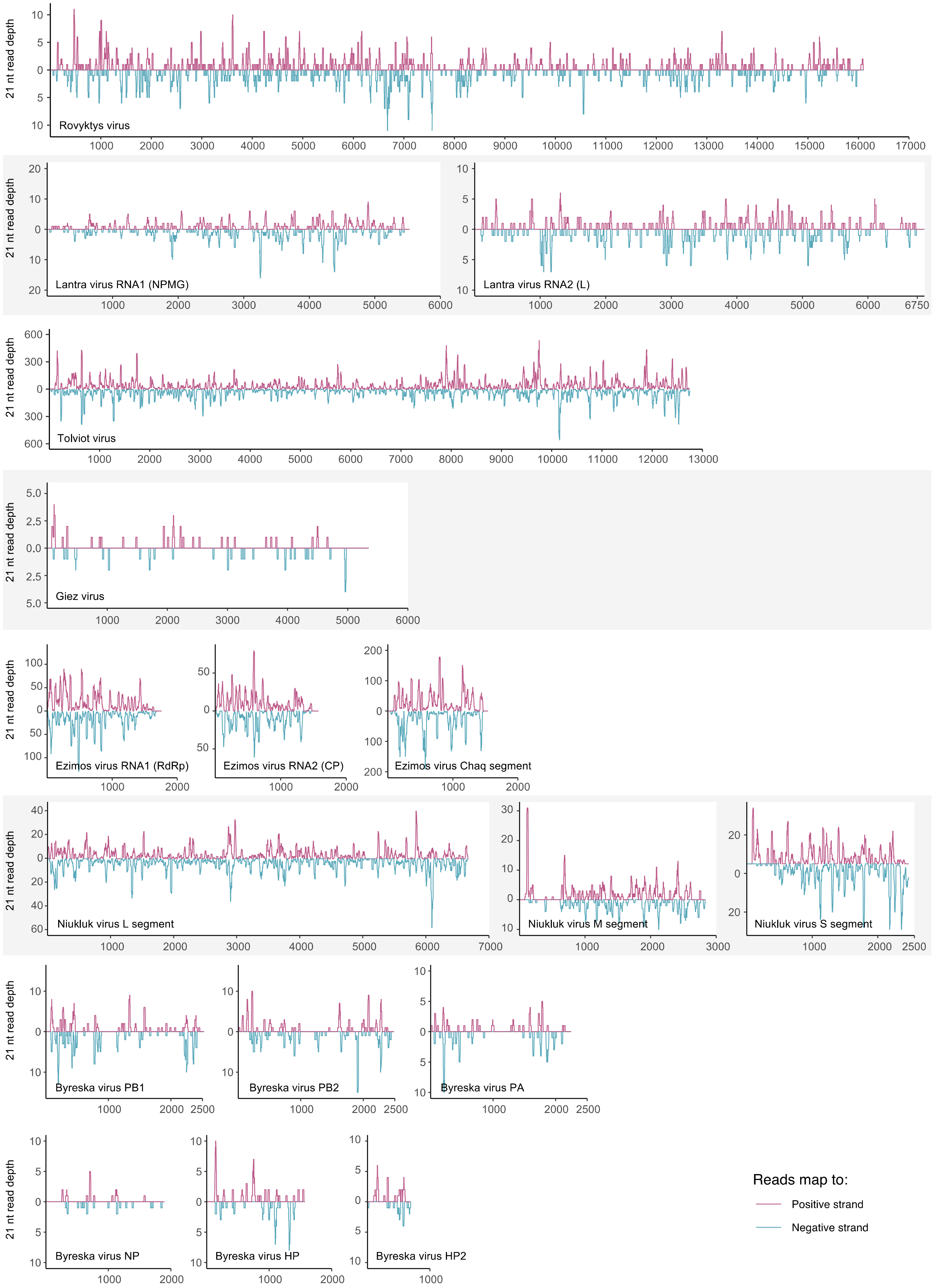
21 nt read depth
Rovyktys virus
Lantra virus RNA1 (NPMG)
Lantra virus RNA2 (L)
Tolviot virus
Giez virus
Ezimos virus RNA1 (RdRp)
Ezimos virus RNA2 (CP)
Ezimos virus Chaq segment
Niukluk virus L segment
Niukluk virus M segment
Niukluk virus S segment
Byreska virus PB1
Byreska virus PB2
Byreska virus PA
Byreska virus NP
Byreska virus HP
Byreska virus HP2
Reads map to:
Positive strand
Negative strand

Figure S4. Potential reassortment or rate variation among Niukluk virus genome segments. Model support for potential breakpoint positions identified by GARD recombination analysis using a concatenated alignment of Niukluk virus genes encoded on different segments. The alignment included 19 sequences and 3081 total sites with no missing data. B) A scaled map of the input alignment showing junction positions between genome segment. One of the two breakpoints identifies the alignment junction between Gc gene (M segment) and the N gene (S segment) at position 2134. The other occurs between the GnGc gene, encoded entirely within the M segment. Δc-AIC from the null model (82.0614) supported multiple breakpoint positions, but Δc-AIC from the single-tree multipartition model (3.37272) did not confidently support topological congruence (i.e. reassortment and/or recombination) over rate variation.

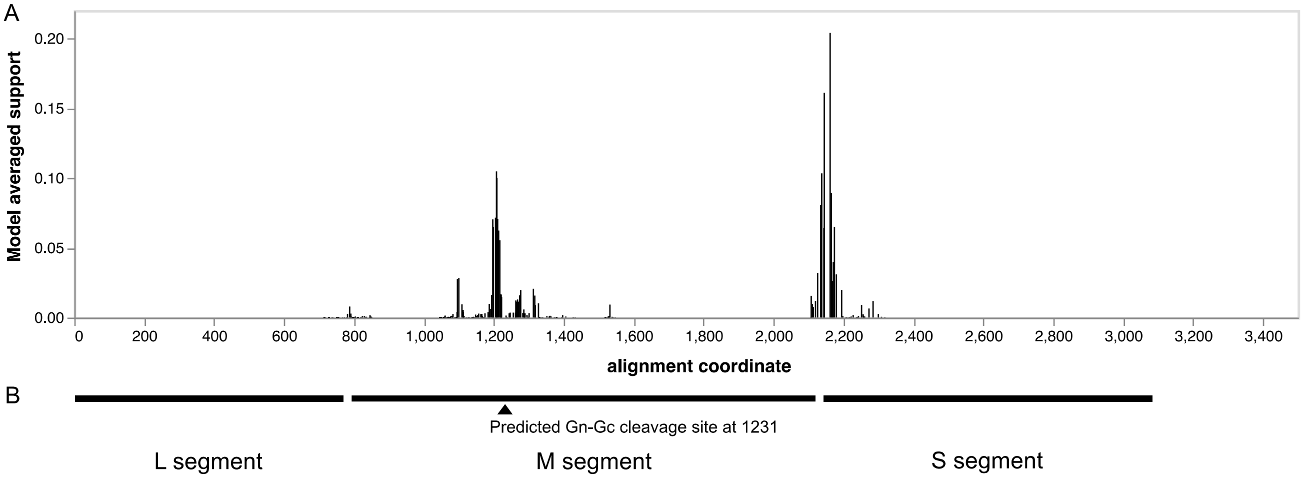

Figure S5. Absence of piwi signatures in virus-derived 24-31 nt reads. Relative nucleotide frequencies at positions 1-12 of putative piRNAs (24-31 nt reads) mapped from ovaries (eggs for Tolviot virus) to *Chaoborus* virus RNAs. Only RNA populations consisting of more than 100 total mapped reads were examined. A color legend is shown to the left of each plot. Nucleotide positions labeling the X axis emphasize two positions at which nucleotide frequency biases associated with the piRNA pathway are known, i.e. U1 and A10.

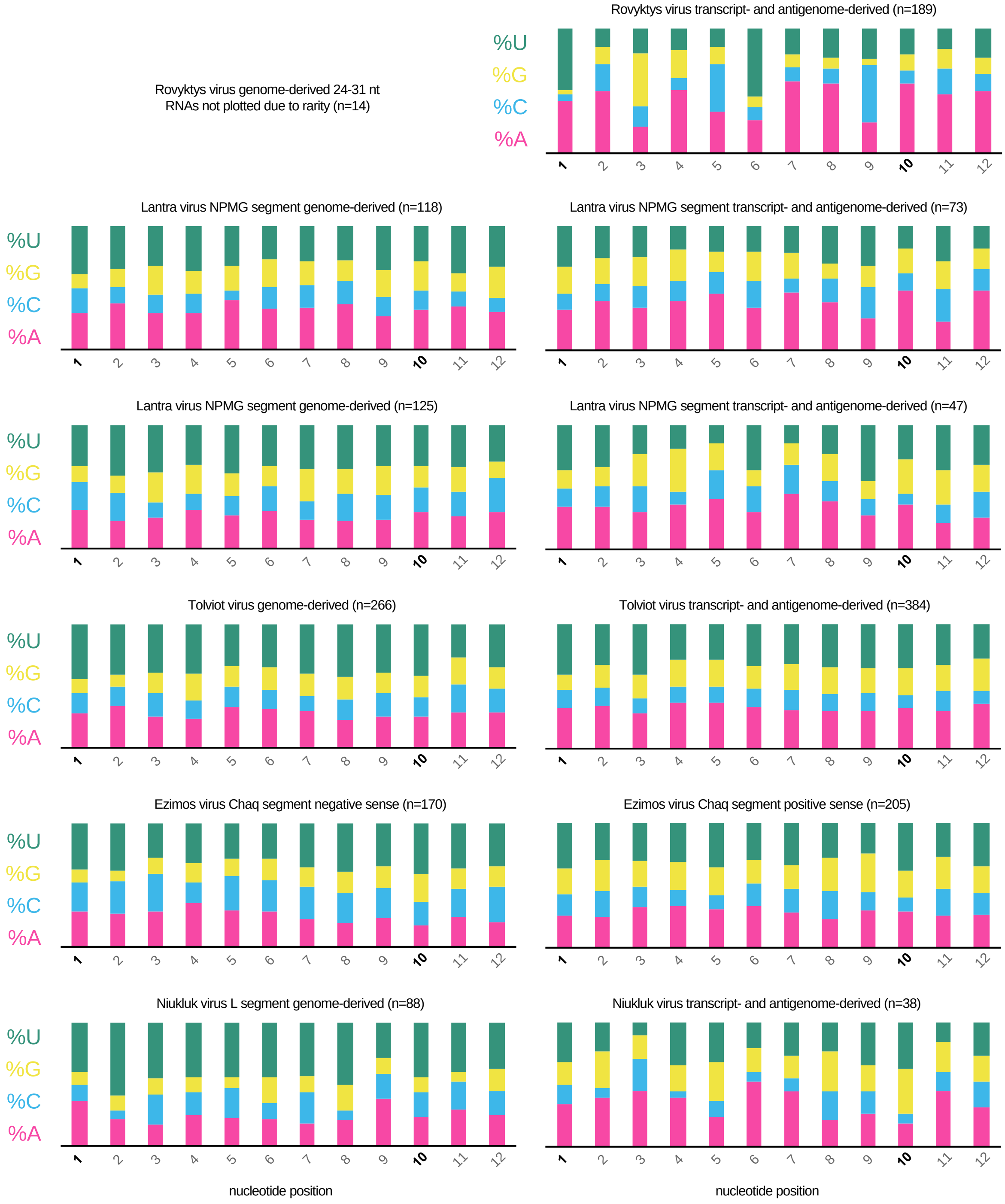

Figure S6. Spatial distribution and abundance of putative PIWI-interacting RNAs derived from a *Chaoborus* virus RNA. The spatial distribution of mapped 24-31 nt piRNAs is shown for the Niukluk virus S segment. The genome maps are displayed under the X axis in negative sense. Arrows indicate open reading frame directionality and are labeled with gene names.

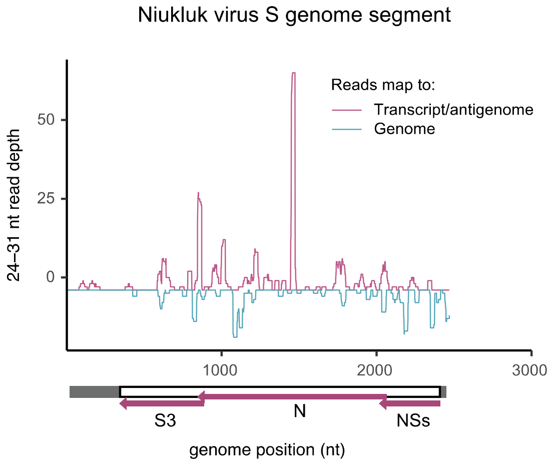

Figure S7. Small RNA populations mapped to putative *Chaoborus* EVEs exhibit piRNA pathway sequence signatures. Relative nucleotide frequencies at positions 1-12 of putative piRNAs (24-31 nt reads) mapped from ovaries to *Chaoborus* EVEs. A color legend is shown to the left of each plot. Nucleotide positions labeling the X axis emphasize two positions at which nucleotide frequency biases associated with the piRNA pathway are known, i.e. U1 and A10.

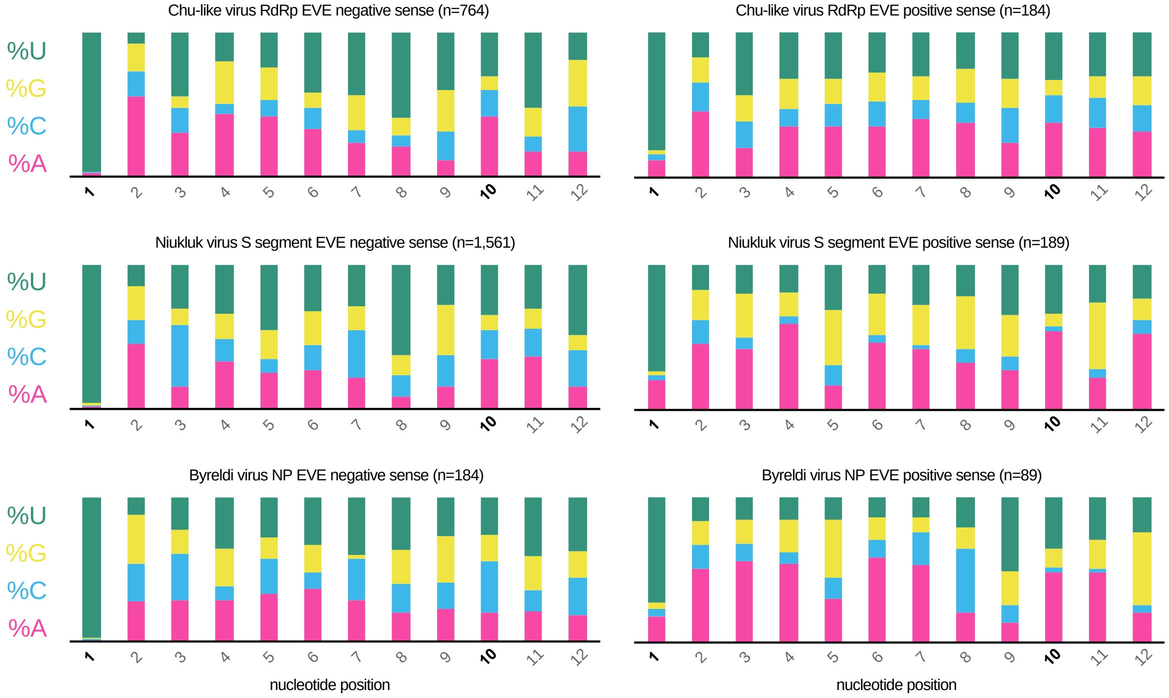

Figure S8. Photograph of intact ovaries cleanly dissected from a female adult *Chaoborus americanus* in Ringer's solution.

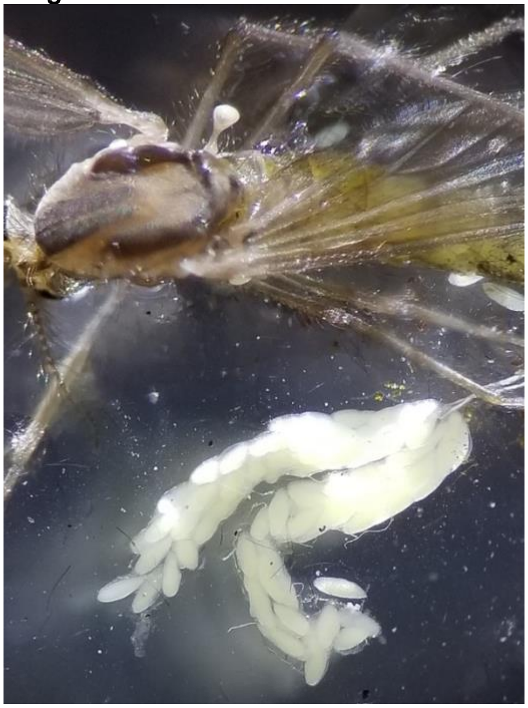

Text S1. Command line software parameters used in this study.

```
#Total RNA assembly
module load Trinity/2.8.4
Trinity --seqType fq --max_memory 100G --left SNO1_R1.fq.gz --right SNO1_R2.fq.gz --CPU 12 --output
Camer_SNO1_Trinity
```

```
#Small RNA trimming
module load BBMap/38.35
bbduk.sh in=smRNA_SNO1_IN.fastq.gz out=smRNA_SNO1_clean.fastq.gz
literal=AGATCGGAAGAGCACACGTCT ktrim=r
```

```
#Small RNA mapping
module load BBMap/38.35
bbmap.sh in=smRNA_SNO1_clean.fastq.gz ref=virus_ref.fasta covstats=covstats_smRNA_SNO1.txt fast
minratio=0.95 outm=mapped_reads_smRNA_SNO1.fastq.gz
```

```
#Small RNA length histograms
module load BBMap/38.35
bbmap.sh in=mapped_reads_smRNA_SNO1_21.fastq.gz lhist=lenhist_smRNA_SNO1.txt
```

```
#Small RNA mapping to bam output
module load BBMap/38.35
module load samtools/1.9
bbmap.sh in=smRNA_SNO1_21nt.fastq.gz ref=virus_ref.fasta fast minratio=0.95
outm=mapped_reads_SNO1_21.bam
```

```
#Stranded coverage depth data
module load bedtools/2.29
bedtools genomecov -ibam mapped_reads_SNO1_21.bam -d -strand + >
virus_ref_SNO1_21nt_bedCov_pos.txt
```

```
#Filter small RNAs by size to extract 21, 18-31, or 24-31 nt reads
module load BBMap/38.35
reformat.sh in=smRNA_SNO1_clean.fastq.gz maxlen=31 minlen=18 out=smRNA_SNO1_18-31nt.fastq.gz
```
